# Outdoor Play as a Mitigating Factor in the Association Between Screen Time for Young Children and Neurodevelopmental Outcomes

**DOI:** 10.1001/jamapediatrics.2022.5356

**Published:** 2023-01-23

**Authors:** Mika Sugiyama, Kenji J. Tsuchiya, Yusuke Okubo, Mohammad Shafiur Rahman, Satoshi Uchiyama, Taeko Harada, Toshiki Iwabuchi, Akemi Okumura, Chikako Nakayasu, Yuko Amma, Haruka Suzuki, Nagahide Takahashi, Barbara Kinsella-Kammerer, Yoko Nomura, Hiroaki Itoh, Tomoko Nishimura

**Affiliations:** 1 United Graduate School of Child Development, Osaka University, Kanazawa University, Hamamatsu University School of Medicine, Chiba University, and University of Fukui, Suita, Japan; 2 Research Center for Child Mental Development, Hamamatsu University School of Medicine, Hamamatsu, Japan; 3 Department of Social Medicine, National Center for Child Health and Development, Tokyo, Japan; 4 Rupiro, the Center for Consultation of Child Development, Hamamatsu, Japan; 5 Department of Child and Adolescent Psychiatry, Nagoya University Graduate School of Medicine, Nagoya, Japan; 6 Queens College and Graduate Center, City University of New York, New York; 7 Department of Psychiatry, Icahn School of Medicine at Mount Sinai, New York; 8 Department of Obstetric and Gynecology, Hamamatsu University School of Medicine, Hamamatsu, Japan

## Abstract

**Importance:**

Whether the association between higher screen time in infancy and later suboptimal neurodevelopment can be mitigated by frequency of outdoor play is unknown.

**Objective:**

To investigate whether higher screen time at age 2 years is associated with neurodevelopmental outcomes at age 4 years and whether this association is mediated by frequency of outdoor play at age 2 years 8 months.

**Design, Setting, and Participants:**

Participants were a subsample of the Hamamatsu Birth Cohort Study for Mothers and Children (HBC Study, N = 1258). Children were born between December 2007 and March 2012 and followed up from 1 year 6 months to 4 years. The analysis was conducted from April 2021 to June 2022.

**Exposures:**

Screen time longer than 1 hour a day at age 2 years was coded as higher screen time.

**Main Outcomes and Measures:**

Standardized scores for communication, daily living skills, and socialization domains of the Vineland Adaptive Behavior Scale, second edition, at age 4 years were used (mean [SD], 100 [15]). The mediating factor was frequency of outdoor play at age 2 years 8 months, with 6 or 7 days per week coded as frequent outdoor play.

**Results:**

Of 885 participants, 445 children (50%) were female; mean (SD) screen time per day was 2.6 (2.0) hours. Causal mediation analyses revealed that higher screen time at age 2 years was associated with lower scores in communication at age 4 years (nonstandardized coefficient *b* = −2.32; 95% CI, −4.03 to −0.60), but the association was not mediated by frequency of outdoor play. Higher screen time was also associated with lower scores in daily living skills (*b* = −1.76; 95% CI, −3.21 to −0.31); 18% of this association was mediated by frequency of outdoor play. Frequency of outdoor play was associated with socialization (*b* = 2.73; 95% CI, 1.06 to 4.39), whereas higher screen time was not (*b* = −1.34; 95% CI, −3.05 to 0.36).

**Conclusions and Relevance:**

Higher screen time at age 2 years was directly associated with poorer communication at age 4 years. It was also associated with daily living skills, but frequency of outdoor play at age 2 years 8 months alleviated it, suggesting outdoor play mitigated the association between higher screen time and suboptimal neurodevelopment. Future research should specify the nature of the associations and intervention measures, enabling targeted interventions that reduce the potential risk in screen time.

## Key Points

**Question:**

Is higher screen time in infancy associated with suboptimal neurodevelopment (communication, daily living skills, and socialization) at age 4 years, and are the associations mediated by frequency of outdoor play?

**Findings:**

In this cohort study, higher screen time (>1 hour a day) at age 2 years was associated with both lower communication and daily living skills at age 4 years. For daily living skills, 18% of the association was mediated and alleviated by the frequency of outdoor play at age 2 years 8 months.

**Meaning:**

Frequent outdoor play may mitigate the connection between higher screen time and later suboptimal neurodevelopment, implying potential for intervention.

## Teaser

This cohort study analyzes data for young children to investigate the association of higher screen time with neurodevelopmental outcomes and whether this association is mediated by outdoor play.

## Introduction

Screen time refers to the amount of time spent watching or using screen devices, such as televisions, video game systems, tablets, and smartphones. The inverse association of screen time with children’s neurodevelopmental well-being has been investigated, with a recent meta-analysis revealing that 75% of children younger than 2 years use them.^1^ These young children are particularly at increased risk of delayed language development,^2,3^ inattention problems,^4^ emotional problems, defiant behaviors,^5^ and poorer reading and academic performance through preschool ages and childhood.^6,7^ This is alarming because the age of first use is becoming lower.^8,9^ Guidelines have been issued by health bodies for caregivers on how to avoid the use of screen devices for children younger than 18 months or to limit screen time to 1 hour daily for children younger than 2 years.^10,11^ However, adherence to these guidelines is low.^1^

Findings on the association of screen time in young children with neurodevelopmental outcomes are still inconclusive. Pagani et al^7^ reported that screen time at ages 6 months and 1 year was moderately associated with cognitive dysfunction in school at age 10 years. However, another study found no association between screen time at ages 6 months, 1 year, or 2 years and cognitive skills at age 3 years.^12^ One issue is that neurodevelopmental domains are different between studies. Some focused on developmental milestones,^13^ language skills,^2,3,14^ or socioemotional problems,^5,15^ while recent studies have suggested that screen time is associated with developmental disorders, such as autism spectrum disorder (ASD).^16,17^ Further, the directionality of the association between high screen time and suboptimal neurodevelopment has been questioned,^18^ although recent studies support directionality of the association.^6,13,15^

Further investigations on the association between screen time and neurodevelopment have examined how it may be related to third factors, including outdoor play. A recent study showed associations of outdoor play with both screen time and preschoolers’ social behavior,^19^ suggesting that the suboptimal neurodevelopmental outcomes of higher screen time in younger children may be mediated by the amount of outdoor play. Outdoor activities have been inversely associated with sedentary time^20^ and screen time^21^ and associated with a child’s cognitive, social, and emotional skills later in life.^22^ The recent COVID-19 pandemic led to children having higher screen time, less outdoor play, and lower physical activity levels,^23,24,25^ putting them at a potentially increased risk for neurodevelopmental problems. What is concerning is that data show screen time has not decreased after seclusion measures were lifted.^26^ Given the data on the pandemic, its aftermath, and the existing cohort, it should be possible to consider how to minimize the effect of higher screen time in a future study.

In this article, we examined whether (1) screen time at age 2 years is associated with neurodevelopmental outcomes at age 4 years; (2) frequency of outdoor play at age 2 years 8 months is associated with neurodevelopmental outcomes at age 4 years; and (3) frequency of outdoor play at age 2 years 8 months mediates the associations between screen time at age 2 years and neurodevelopmental outcomes. Using the second edition of the Vineland Adaptive Behavior Scale (VABS-II),^27^ we measured neurodevelopmental outcomes that could capture communication, daily living skills, and socialization domains.

## Methods

### Study Design

This study was conducted as a part of an ongoing birth cohort study, the Hamamatsu Birth Cohort Study for Mothers and Children (HBC Study, N = 1258). We invited all pregnant women who visited the 2 research sites and gave birth between December 2007 and March 2012 to join the study. Further details of the procedures are described elsewhere.^28,29^ This study was conducted under the Strengthening the Reporting of Observational Studies in Epidemiology (STROBE) reporting guidelines.

### Participants

We extracted data for a subsample of children participating in the study who completed observations at ages 1 year 6 months, 2 years, 2 years 8 months, and 4 years; children missing 1 or more observations were excluded. We compared the participants (n = 885) and the excluded children (n = 373) (eTable 1 in Supplement 1). This study was approved by the institutional review board of Hamamatsu University School of Medicine. Written informed consent was obtained from all caregivers for their own and their child’s participation.

### Measurement

#### Screen Time (Exposure of Interest)

We interviewed the parents of the participating children about their children’s lifestyle at age 2 years using 1 item from the ISAAC Phase 3 Environmental Questionnaire, developed for the International Study of Asthma and Allergies in Childhood (ISAAC)^30^ to measure screen time. Screen time was defined as mean hours of screen time per day watching or using television, DVD, video, internet and mobile phone, or video games, both actively and passively.^10^ ISAAC Phase 3 started in the early 2000s, and the original item asked, “During a normal week, how many hours a day does your child watch TV?” We amended this to describe further, “TV here includes DVD, video, internet, mobile phones and video games, regardless of active or passive viewing.” In our analyses, we dichotomized the variable, with 1 indicating longer than 1 hour a day (higher screen time) and 0 representing 1 hour or less. The cutoff was consistent with the published guideline by the American Academy of Pediatrics,^10^ which recommended 1 hour a day of noneducational viewing for children aged 2 to 5 years.

#### Outdoor Play (Mediator of Interest)

Parents were asked 1 question from the ISAAC questionnaire when children were aged 2 years 8 months to ascertain the frequency and duration of outdoor play. The original question, “How many days during a normal week does your child go and stay outside for 30 minutes or longer to make him/her breathe hard?” was modified to specify the frequency of physical activity when outdoors. In the analyses, we dichotomized this variable, with 1 indicating fewer than 6 days with 30 minutes or longer of any type of outdoor play a week (ie, infrequent outdoor play) and 0 for 6 or 7 days. We had to dichotomize this variable because the original value was heavily skewed, with a median of 6 days a week, and also because commands for causal mediation analyses available in the software, Stata version 17.0 (StataCorp), only allowed mediators regressed on exposure variables in linear or logistic functions.

#### Neurodevelopmental Outcomes

We adopted the Japanese version^31^ of VABS-II^27^ to measure the 3 domains of children’s neurodevelopmental outcomes we analyzed: communication (receptive, expressive, and written language skills), daily living skills (skills entailing personal, domestic, and community life), and socialization (interpersonal relationships, play, and coping skills). Assessment was made through a semistructured parental interview, which allowed us to obtain age-adjusted V scores, with a mean (SD) of 100 (15).

#### Covariates

To select appropriate covariates, we drew directed acyclic graphics using DAGitty software.^32^ Variables that could cause any 2 of 3 (screen time, outdoor play, or neurodevelopmental outcomes) were treated as potential confounders and entered into the analyses. We treated child’s sex, maternal and paternal education, and ASD symptoms at age 1 year 6 months as covariates in the adjusted model (Figure). Symptoms of ASD were measured using the Modified Checklist for Autism in Toddlers^33^ (Japanese version^34^) at age 1 year 6 months. The cutoff for a possible early diagnosis of ASD was set at either 3 or more points for a total score or 2 or more points for 10 critical items as previously developed in Japan.^35^ We assigned a value of 1 if the child scored on or above the cutoff and 0 otherwise.

**Figure.**
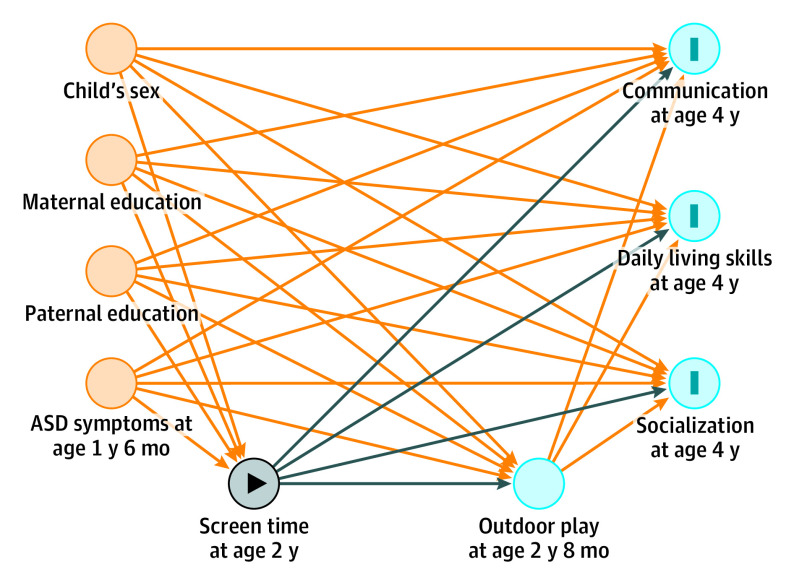
Path Diagrams of Screen Time at Age 2 Years, Outdoor Play at Age 2 Years 8 Months, and Neurodevelopmental Outcomes (Communication, Daily Living Skills, and Socialization) at Age 4 Years ASD indicates autism spectrum disorder; dark blue lines, causal paths; orange lines, biasing paths; gray circle, exposure variable; blue circles with I, outcome variables; blue circle without I, ancestor of outcomes; orange circles, ancestors of exposure.

### Analysis

First we independently tested the associations of screen time at age 2 years and outdoor play at age 2 years 8 months with communication, daily living skills, and socialization at age 4 years using linear regression in the crude and adjusted models.

In the causal mediation analyses, we investigated whether outdoor play mediated the association between screen time and neurodevelopmental outcomes (the 3 domains) using the paramed command^36,37^ in Stata. Causal mediation analyses, originally proposed by Robins and Greenland,^38^ allow statistical models to have an interaction term for an exposure and mediator and decompose total effects into natural direct effect (NDE) and natural indirect effect (NIE) under a counterfactual framework. To achieve parsimony and ease model interpretation, we excluded the interaction terms from the statistical models in the mediation analyses if the exclusion did not meaningfully change either of the effect estimates (NDE and NIE) by more than 10%. When an interaction term is removed, the 2-way decomposition of controlled direct effect and NIE originally proposed by Baron and Kenny^39^ is mathematically equivalent to the 2-way decomposition of NDE and NIE proposed by Robins and Greenland.^38^ Accordingly, NDE is the difference in counterfactual outcomes between the presence and absence of the exposure if the mediator is held at the level in the absence of exposure. NIE is the difference in counterfactual outcomes between the presence and absence of the mediator if the exposure is held present. Total effect corresponds to a sum of NDE and NIE.

We conducted sensitivity analyses to see if the finding was replicated in subsamples of children whose outdoor play was assessed during the warm months at our site (April-November, mean monthly temperature ≥10 °C) and during the cold months. If the analyzed results were inconsistent, we repeated the causal mediation analyses of the final model, adjusting for the available covariates and the season of the outdoor play measurement (warm vs cold months).

Effect estimates were reported based on both unadjusted (crude) and adjusted models. Standard errors and corresponding 95% CIs (bias-corrected) were estimated using bootstrapping procedures with 100 replications. The cutoff for 2-sided *P* values was set at .05.

## Results

Table 1 presents the characteristics of the 885 participants. We also compared their baseline data with those of the nonparticipating children in the original cohort (n = 373) (eTable 1 in Supplement 1). More boys (56% vs 50%) were included, and the level of maternal education (13.9 vs 13.6 years) was higher in the participants than in the nonparticipants. No other significant difference was found.

**Table 1.** Characteristics of Study Participants (n = 885)

Characteristic	No. (%)				
	All participants	Screen time 0-1 h at age 2 y		Screen time >1 h at age 2 y	
		Outdoor play ≥6 d/wk at age 2 y 8 mo	Outdoor play <6 d/wk at age 2 y 8 mo	Outdoor play ≥6 d/wk at age 2 y 8 mo	Outdoor play <6 d/wk at age 2 y 8 mo
No. of participating children	885	177	88	301	319
Sex					
Female	445 (50)	92 (52)	48 (55)	136 (45)	169 (53)
Male	440 (50)	85 (48)	40 (45)	165 (55)	150 (47)
Parity, first born	434 (49)	92 (52)	47 (53)	136 (45)	159 (50)
Maternal education, mean (SD), y	13.9 (1.9)	14.4 (1.9)	14.2 (1.9)	14.0 (1.9)	13.6 (1.7)
Paternal education, mean (SD), y	14.2 (2.6)	14.2 (2.7)	14.5 (2.4)	14.1 (2.5)	14.1 (2.8)
ASD symptoms at age 1 y 6 mo^a^	175 (20)	28 (16)	18 (20)	56 (19)	73 (23)
Screen time at age 2 y, mean (SD), h/d	2.6 (2.0)	0.8 (0.4)	0.8 (0.3)	3.1 (1.6)	3.5 (2.1)
Outdoor play at age 2 y 8 mo, mean (SD), d/wk^b^	5.1 (2.2)	6.9 (0.3)	3.4 (1.5)	6.9 (0.4)	3.0 (1.5)
VABS-II domain score at age 4 years, mean (SD)					
Communication	99.2 (13.0)	101.1 (11.9)	101.8 (13.8)	99.0 (12.5)	97.7 (13.6)
Daily living skills	100.6 (10.4)	102.5 (9.6)	101.2 (11.4)	101.0 (10.7)	98.9 (10.1)
Socialization	103.1 (12.5)	105.2 (11.8)	103.0 (11.6)	104.1 (11.9)	101.0 (13.3)

Abbreviations: ASD, autism spectrum disorder; VABS-II, second edition of the Vineland Adaptive Behavior Scale.

^a^ ASD symptoms were measured using the Modified Checklist for Autism in Toddlers (Japanese version) at age 1 year 6 months. The cutoff for ASD symptoms was set at either ≥3 points for a total score or ≥2 points for 10 critical items as previously developed in Japan. We assigned a value of 1 if the child scored on or above the cutoff and 0 otherwise.

^b^ Number of days of ≥30 minutes of any type of outdoor play a week, dichotomized into ≥6 (reference) and <6 (infrequent outdoor play).

### Independent Associations of Screen Time and Outdoor Play With the 3 Domains of Neurodevelopment

In the direct-only, crude models (Table 2), higher screen time (>1 hour a day) at age 2 years was significantly and inversely associated with communication (nonstandardized coefficient *b* = −3.05; 95% CI, −4.91 to −1.19), daily living skills (*b* = −2.13; 95% CI, −3.62 to −0.65), and socialization (*b* = −1.97; 95% CI, −3.74 to −0.21) at age 4 years. The associations remained significant after covariate adjustment except for socialization (*b* = −1.34; 95% CI, −3.05 to 0.36). Infrequent outdoor play (<6 days a week) at age 2 years 8 months was not associated with communication but was significantly and inversely associated with daily living skills (*b* = −2.16; 95% CI, −3.55 to −0.77) and socialization (*b* = −3.03; 95% CI, −4.71 to −1.35). These associations remained significant in the adjusted models (Table 2).

**Table 2.** Independent Associations of Screen Time at Age 2 Years and Outdoor Play at Age 2 Years 8 Months With the 3 Domains of Neurodevelopmental Outcomes (Vineland Adaptive Behavior Scale Scores) at Age 4 Years (n = 885)

	Neurodevelopmental outcomes, *b* (95% CI)		
	Communication	Daily living skills	Socialization
**Screen time >1 h/d at age 2 y (higher screen time)^a^**			
Crude	−3.05 (−4.91 to −1.19)	−2.13 (−3.62 to −0.65)	−1.97 (−3.74 to −0.21)
Adjusted^b^	−2.32 (−4.03 to −0.60)	−1.76 (−3.21 to −0.31)	−1.34 (−3.05 to 0.36)
**Outdoor play <6 d/wk at age 2 y 8 mo (infrequent outdoor play)^c^**			
Crude	−1.23 (−3.00 to 0.53)	−2.16 (−3.55 to −0.77)	−3.03 (−4.71 to −1.35)
Adjusted^b^	−1.18 (−2.85 to 0.47)	−2.07 (−3.45 to −0.68)	−2.73 (−4.39 to −1.06)

Abbreviations: ASD, autism spectrum disorder; *b*, nonstandardized path coefficient.

^a^ Screen time was the mean number of hours spent each day watching or using television, DVD, video, internet and mobile phone, or video games, both actively and passively; dichotomized into 0-1 hour (reference) and >1 hour (higher screen time).

^b^ Sex of the child, maternal and paternal education, and ASD symptoms at age 1 year 6 months were included as covariates in the adjusted model.

^c^ Number of days of ≥30 minutes of any type of outdoor play a week, dichotomized into ≥6 (reference) and <6 (infrequent outdoor play).

### Association Between Screen Time and Outdoor Play

Higher screen time at age 2 years was significantly associated with infrequent outdoor play at age 2 years 8 months after covariate adjustment (odds ratio, 2.03; 95% CI, 1.48-2.76).

### Causal Mediation Analyses

We first checked whether exposure-mediator interactions should be included by comparing the NDE and NIE estimates with and without the interaction term. Because the estimates did not show a change greater than 10%, we omitted the interaction terms and decomposed the associations.^38^

For the outcome communication, a large total effect (*b* = −2.32; 95% CI, −3.61 to −0.49) and NDE (*b* = −2.17; 95% CI, −3.48 to −0.23) and a nonsignificant, smaller NIE (*b* = −0.15; 95% CI, −0.48 to 0.16) were found (Table 3). For daily living skills, a significant total effect (*b* = −1.76; 95% CI, −3.57 to −0.47), a nonsignificant NDE (*b* = −1.44; 95% CI, −2.71 to 0.11), and a significant NIE (*b* = −0.32; 95% CI, −0.77 to −0.06) were found, with 18.1% mediated. For socialization, no significant total effect was found.

**Table 3.** Causal Mediation Analyses Showing the Decomposition of Associations of a Higher Screen Time at Age 2 Years With the 3 Domains of Neurodevelopmental Outcomes (Vineland Adaptive Behavior Scale Scores) at Age 4 Years (n = 885)^a^

	Neurodevelopmental outcomes, *b* (95% CI)^b^		
	Communication	Daily living skills	Socialization
Natural direct effect^c^	−2.17 (−3.48 to −0.23)	−1.44 (−2.71 to 0.11)	−0.91 (−2.58 to 0.89)
Natural indirect effect^c^	−0.15 (−0.48 to 0.16)	−0.32 (−0.77 to −0.06)	−0.44 (−0.80 to −0.19)
Total effect	−2.32 (−3.61 to −0.49)	−1.76 (−3.57 to −0.47)	−1.35 (−3.01 to 0.48)

Abbreviations: ASD, autism spectrum disorder; *b*, nonstandardized path coefficient; NDE, natural direct effect; NIE, natural indirect effect.

^a^ Screen time was the mean number of hours spent each day watching or using television, DVD, video, internet and mobile phone, or video games, both actively and passively; dichotomized into 0-1 hour (reference) and >1 hour (higher screen time).

^b^ Sex of the child, maternal and paternal education, and ASD symptoms at age 1 year 6 months were included as covariates.

^c^ NDE (bypassing outdoor play at age 2 years 8 months) vs NIE (via outdoor play at age 2 years 8 months). The coefficients for NDE reflect the estimated difference in the domain score between children with screen time >1 hour and those with screen time of ≤1 hour given that the frequency of outdoor play was fixed at 6-7 days a week. The coefficients for NIE reflect the estimated difference in the domain score between children with a frequency of outdoor play of 6-7 days and those with <6 days a week given that screen time was fixed at >1 hour a day.

In the sensitivity analyses, the direction and significance of the associations were supported in children whose outdoor play was assessed during warm months but not in those with outdoor play assessed during cold months (eTable 2 in Supplement 1). To remove potential bias associated with seasonal temperature changes,^40^ we iterated the final analysis, adjusting for the available covariates and the season (1 = warm, 0 = cold), where the direction and significance of the associations remained the same as the results in Table 3.

## Discussion

This study showed that a higher screen time (>1 hour a day) at age 2 years was associated with poorer neurodevelopmental outcomes at age 4 years. Major findings were as follows: (1) Screen time at age 2 years was associated with communication and daily living skills; (2) the frequency of outdoor play did not mediate the association for communication, but it did mediate the association for daily living skills as indicated by a nonsignificant NDE but significant NIE and total effect, with the proportion of mediation 18%; (3) screen time at age 2 years was not significantly associated with socialization, but frequent outdoor play was. These associations were upheld after adjusting for potential confounders, including child’s sex, parental education, and child’s ASD symptoms at age 1 year 6 months.

Tomopoulos et al^2^ demonstrated that screen time in children as young as 6 months was associated with an increased risk for delay in language development. This observation is supported by recent studies.^5,41,42^ The direct link between early screen time and later language and communication difficulties in these studies is in line with the results of our causal mediation analysis for communication, where total effect and NDE were significant while NIE was nonsignificant, with the coefficient of NIE much smaller than that of NDE, indicating no mediation (Table 3).

Similarly, we found a significant total effect in the association between screen time and daily living skills (Table 3). The coefficient of NDE was directed to minus, as was expected from the literature, but was nonsignificant. Notably, a significant NIE suggests that the association between screen time and daily living skills is mediated at least partly by a change in the frequency of outdoor play. Specifically, a higher screen time at age 2 years decreased the score of daily living skills at age 4 years, although the decrease in the score may be reduced by 18% with a change in the frequency of outdoor play at 2 years 8 months, from less than 6 days to 6 and 7 days a week.

As expected, we found that the coefficient of total effect as a sign of association between screen time and socialization was negative, although nonsignificant. The literature offers limited support for an association between screen time and socialization: Some studies support a significant assciation^19,43,44^; others do not.^12,42^ In contrast, frequency of outdoor play is consistently associated with socialization. Similarly, consistent with the literature,^7,24^ higher screen time is associated with frequent outdoor play. Intriguingly, outdoor play is associated only with daily living skills and socialization and not communication (Table 2). Because an increase in outdoor play is correlated inversely with sedentary behaviors and positively with physical activity in young children,^20,45^ understanding the role of outdoor play in the link between early screen time and later neurodevelopment may offer leads for possible interventions.

Parental education and ASD symptoms are potential cofounders that need to be considered. Both are associated with changes in screen time^18,46^ and are also determinants of neurodevelopmental outcomes.^47,48^ We observed a reduction in both coefficients after adjustment (Table 2). As they were expected to confound the association between outdoor play and neurodevelopmental outcomes,^49,50^ we tested for exposure-outcome association and mediator-outcome association, both showing that confounding did not fully explain the associations.

Different mechanisms that might explain a direct association between screen time and neurodevelopment in communication have been suggested. One study suggested that an increase in audible time to television decreased the magnitude of attention to parental vocalization, leading to a decrease in child vocalization.^51^ Another study supported this, suggesting that background television viewing might decrease parent-child interaction.^52^ These associations have been observed in young children with ASD, who are prone to delays in language skill development.^53^ Specifically, the amount of passive screen viewing has been inversely associated with the receptive language score of children with ASD at ages 1 to 3 years,^42^ suggesting that high screen time in young children is associated with suboptimal neurodevelopment in communication, possibly through reduced attention to adults and compromised comprehension in conversation, and that such a explanation stands irrespective of levels of language skills or ASD diagnosis. However, understanding the association of screen time with daily living skills and socialization is complex and needs to take account of cofounders such as the cumulative risk of socioeconomic disadvantage and genetics, dating back to birth or earlier.

A prospective study has suggested that screen time, together with parenting attitude, can function as a mediator between early socioeconomic disadvantage and distal outcomes, including socioemotional suboptimality.^54^ It is also possible that the time spent in outdoor activities could mediate the relationship between parenting attitude and screen time.^55,56,57^ Taken together, it is imperative to investigate the role of outdoor play in optimal child neurodevelopment, not limited to potential remedial and mitigating factors. For example, recent studies have indicated the relevance of “green time”^58^ and sleep^59^ when considering the role of outdoor play in the triangulation of screen time, parenting attitude, and socioemotional neurodevelopment. All these factors need to be reevaluated in the face of natural disasters such as a pandemic.

### Strengths and Limitations

This study has several advantages. First, it is a longitudinal study, with a relatively large sample size. Second, causal mediation analysis allowed us to assess the mediating effect of outdoor play in the association between screen time and neurodevelopment, suggesting a potential point for intervention. However, it also has limitations. The use of parental reports to measure screen time may have resulted in underestimation. Information about the types of screen programs watched and played is lacking; this should have been collected because the effect of high screen time differs depending on the type of program.^5,60^ Concerning the generalizability of the findings, children raised in the early 2010s in this study might not have been exposed to screen time as much as those raised in the 2020s. Ownership of digital devices, including mobile phones and personal computers, was stable, at around 95%, during the 2010s, and watching YouTube movies had been increasingly prevalent in Japan since the 2011 Great East Japan Earthquake.^61^

## Conclusions

In this cohort study, higher screen time at age 2 years was associated with suboptimal neurodevelopment in communication at age 4 years, and this association was not confounded or mediated by factors examined in this study. Higher screen time at age 2 years was associated with suboptimal development in daily living skills at age 4 years and mediated by outdoor play at age 2 years 8 months. Future research should specify the nature of the associations and intervention measures to reduce the potential risk inherent in higher screen time and explore the mechanisms underlying the association between screen time and neurodevelopmental outcomes. Further, updating guidelines regarding media use is extremely important for parents, educators, researchers, and the children themselves.

## Notes

Supplement 1. **eTable 1.** Characteristics of children excluded from the analysis (n = 373) in comparison with children included in the analysis (n = 885) **eTable 2.** Causal mediation analyses showing the decomposition of the associations between screen time at age 2 and neurodevelopmental outcomes at age 4 in children having outdoor play measured in the warm months, in the cold months, and in all the participating children with a further adjustment for the seasons when outdoor play was measured

Supplement 2. Data sharing statement
