# Supplementary material for: Outdoor Play as a Mitigating Factor in the Association Between Screen Time for Young Children and Neurodevelopmental Outcomes
Source: JAMA Pediatr. 2023 Jan 23;177(3):303–10. doi: 10.1001/jamapediatrics.2022.5356 (PMC9871942; doi:10.1001/jamapediatrics.2022.5356)
Supplement: Supplement 1. — eTable 1. Characteristics of children excluded from the analysis (n = 373) in comparison with children included in the analysis (n = 885) eTable 2. Causal mediation analyses showing the decomposition of the associations between screen time at age 2 and neurodevelopmental outcomes at age 4 in children having outdoor play measured in the warm months, in the cold months, and in all the participating children with a further adjustment for the seasons when outdoor play was measured [file jamapediatr-e225356-s001.pdf]

Supplemental Online Content

Sugiyama M, Tsuchiya KJ, Okubo Y, et al. Outdoor play as a mitigating factor in the association between screen time for young children and neurodevelopmental outcomes. *JAMA Pediatr*. Published online January 23, 2023. doi:10.1001/jamapediatrics.2022.5356

eTable 1. Characteristics of children excluded from the analysis (n = 373) in comparison with children included in the analysis (n = 885)

eTable 2. Causal mediation analyses showing the decomposition of the associations between screen time at age 2 and neurodevelopmental outcomes at age 4 in children having outdoor play measured in the warm months, in the cold months, and in all the participating children with a further adjustment for the seasons when outdoor play was measured

This supplemental material has been provided by the authors to give readers additional information about their work.

© 2023 Sugiyama M et al. *JAMA Pediatr.*

eTable 1. Characteristics of children excluded from the analysis (n = 373) in comparison with children included in the analysis (n = 885).

	**Excluded from the analysis**	**Included in the analysis**	**Statistics**
Number of children	373	885	
Sex of the child (female, %)	446 (50%)	165 (44%)	$\chi2(1)$=3.99, p=.046
Parity (first born, %)	192 (51%)	434 (49%)	$\chi2(1)$=0.62, p=.43
Maternal education (years)	13.6 (SD 2.2)	13.9 (SD 1.9)	t(1256)=2.64, p=.008
Paternal education (years)	13.9 (SD 2.8)	14.2 (SD 2.6)	t(1256)=1.71, p=.09

© 2023 Sugiyama M et al. *JAMA Pediatr.*

eTable 2. Causal mediation analyses showing the decomposition of the associations between screen time at age 2 and neurodevelopmental outcomes (the standardized scores of communication/daily living skills/socialization domains of the Vineland Adaptive Behavior Scale) at age 4 in children having outdoor play measured in the warm months(April–November, n = 585), in the cold months (December–March, n = 300), and in all the participating children with a further adjustment for the seasons when outdoor play was measured (warm vs cold months: n = 885). Natural direct effect (bypassing outdoor play at age 2 years 8 months) vs natural indirect effect (via outdoor play at age 2 years 8 months).

	**Neurodevelopmental outcomes of children having outdoor play, measured in warm months (n=585)**		
	Communication<br>b (95% CI)	Daily living Skills<br>b (95% CI)	Socialization<br>b (95% CI)
Natural direct effect<br>(NDE)	**−2.04<br>(−4.48, −0.35)**	**−2.21<br>(−3.85, −0.91)**	−1.67<br>(−4.05, 0.06)
Natural indirect effect<br>(NIE)	−0.14<br>(−0.73, 0.35)	**−0.36<br>(−0.73, −0.02)**	**−0.34<br>(−1.04, −0.01)**
Total effect<br>(TE)	**−2.18<br>(−4.27, −0.09)**	**−2.57<br>(−3.81, −1.34)**	**−2.01<br>(−4.23, −0.29)**
	**Neurodevelopmental outcomes of children having outdoor play, measured in cold months (n=300)**		
	Communication<br>b (95% CI)	Daily living Skills<br>b (95% CI)	Socialization<br>b (95% CI)
Natural direct effect<br>(NDE)	−2.21<br>(−5.35, 0.32)	0.14<br>(−2.27, 3.06)	0.82<br>(-2.62, 4.37)
Natural indirect effect<br>(NIE)	−0.11<br>(−0.62, 0.12)	−0.21<br>(−0.62, 0.21)	−0.40<br>(−1.00, 0.10)
Total effect<br>(TE)	−2.31<br>(−5.41, 0.21)	−0.07<br>(−2.54, 2.72)	0.43<br>(-2.79, 3.86)
	**Neurodevelopmental outcomes of all the participating children (n=885) adjusted for child's sex, parental education, ASD symptoms and seasons of measuring outdoor play**		
	Communication<br>b (95% CI)	Daily living skills<br>b (95% CI)	Socialization<br>b (95% CI)
Natural direct effect<br>(NDE)	**−2.17<br>(−3.93, −0.41)**	**−1.44<br>(−2.78, -0.17)**	−0.91<br>(−2.62, 0.90)
Natural indirect effect<br>(NIE)	−0.15<br>(−0.47, 0.17)	**−0.34<br>(−0.87, −0.10)**	**−0.44<br>(−0.80, −0.20)**
Total effect<br>(TE)	**−2.32<br>(−3.61, −0.51)**	**−1.78<br>(−3.57, −0.51)**	−1.35<br>(−3.27, 0.32)

Screen time was the mean number of hours spent each day watching or using television, DVD, video, internet and mobile phone, or video games, both actively and passively; dichotomized into 0–1 hour (reference) and >1 hour (higher screen time).

Outdoor play was the mean number of days of >30 minutes of any type of outdoor play a week; dichotomized into ≥6 (reference) and <6 days (infrequent outdoor play).

Sex of the child, maternal and paternal education, and ASD symptoms at age 1 year 6 months were included as covariates.

© 2023 Sugiyama M et al. *JAMA Pediatr.*

b = non-standardized path coefficient. CI = confidence interval

NDE (bypassing outdoor play at age 2 years 8 months) vs NIE (via outdoor play at age 2 years 8 months). The coefficients for NDE reflect the estimated difference in the domain score between children with screen time >1 hour and those with screen time of ≤1 hour given that the frequency of outdoor play was fixed at 6–7 days a week. The coefficients for NIE reflect the estimated difference in the domain score between children with a frequency of outdoor play of 6–7 days and those with <6 days a week given that screen time was fixed at >1 hour a day.

© 2023 Sugiyama M et al. *JAMA Pediatr.*
