# Supplementary material for: Outdoor Play as a Mitigating Factor in the Association Between Screen Time for Young Children and Neurodevelopmental Outcomes
Source: JAMA Pediatr. 2023 Jan 23;177(3):303–10. doi: 10.1001/jamapediatrics.2022.5356 (PMC9871942; doi:10.1001/jamapediatrics.2022.5356)
Supplement: Supplement 2. — Data sharing statement [file jamapediatr-e225356-s002.pdf]

Data Sharing Statement

Sugiyama. Outdoor Play as a Mitigating Factor in the Association Between Screen Time for Young Children and Neurodevelopmental Outcomes. *JAMA Pediatr*. Published January 23, 2023. doi:10.1001/jamapediatrics.2022.5356

Data
Data available: No
